# Joint modelling of left- and interval-censored viral load for couples in Mozambique

**DOI:** 10.1371/journal.pone.0345307

**Published:** 2026-03-30

**Authors:** Adelino J. C. Juga, Niel Hens, Nafissa Osman, Marc Aerts

**Affiliations:** 1 Department of Mathematics and Informatics, Faculty of Sciences, Eduardo Mondlane University, Maputo, Mozambique; 2 Data Science Institute (DSI), I-BioStat, Hasselt University, Hasselt, Belgium; 3 Centre for Health Economic Research and Modelling Infectious Diseases, Vaccine and Infectious Disease Institute (VAXINFECTIO), University of Antwerp, Antwerp, Belgium; 4 Department of Obstetrics and Gynaecology, Central Hospital, Maputo, Mozambique; 5 Faculty of Medicine, Eduardo Mondlane University, Maputo, Mozambique; Reggio Emilia Local Agency - IRCCS Advanced Technologies and Care Models in Oncology: Azienda Unita Sanitaria Locale - IRCCS Tecnologie Avanzate e Modelli Assistenziali in Oncologia di Reggio Emilia, ITALY

## Abstract

Mathematical and statistical models have been essential tools in exploring the dynamics of viral load measures, understanding of the pathogenesis of HIV-1 infection and in assessment of the potency of antiretroviral therapies. However, this can be challenging due to the potential intra-couple correlation as well as the presence of multiple measurements collected from the same study site or cluster. A second complication arises due to censoring of the individual viral load measurements. The aim of this paper is to investigate the association between age and viral load of woman and man within a couple, while accounting for the presence of antiretroviral biomarkers in blood. An additional question of interest is how weakly or strongly are viral load of woman and man correlated, and how does this correlation depend on covariates as well. Joint marginal and random-effects models assuming constant and non-constant correlation were fitted using maximum likelihood while accounting for the left- and interval-censoring nature of the two viral loads. Findings show a weak positive correlation between the viral loads of women and men. Next, interaction between age and antiretroviral biomarker’s presence is only significant in the mean viral load for women, showing that the effect of age on a women’s viral load varies by the antiretroviral biomarker’s status. No age effect on the mean viral load for men is observed. These findings reinforce the need of interventions that stimulate adherence to antiretroviral therapy treatment once one or both partners are HIV infected as well as to monitor their viral load as they get older, especially the female partner.

## 1 Introduction

There has been accumulating evidence from literature showing that antiretroviral therapy (ART) prevents morbidity and mortality for people living with HIV (PLHIV), and also has clear Human Immunodeficiency Virus (HIV) prevention benefits [1–3]. In 2012, the World Health Organization (WHO) endorsed that individuals with HIV in serodiscordant couples should be counselled on the use of ART to reduce HIV transmission in the uninfected partner [4]. With proper adherence to treatment, antiretroviral therapy has the potential to suppress viral replication, often below the level of detection (LOD) by commercially available tests. Studies showed that the risk of transmission is effectively negligible below this level [5–7]. This highlights the importance of viral load (VL) as a marker of treatment efficacy, and has led to a growing consensus that people who have reached and maintained undetectable VL do not transmit HIV sexually to their partners [5,7].

Several factors have been associated with viral load levels and the risk of HIV transmission among serodiscordant couples. Higher VL in the HIV-positive partner is a major predictor of seroconversion in the HIV-negative partner, with transmission risk increasing significantly with higher HIV RNA levels and decreasing markedly with sustained viral suppression through effective antiretroviral therapy (ART) and consistent condom use [8]. Additionally, detectable viral load has been linked to lower likelihood of maintaining serodiscordance, while lack of disclosure of HIV status, younger age, and suboptimal ART adherence are associated with unsuppressed viral loads in positive partners [9].

The pivotal HPTN 052 randomized controlled trial demonstrated that early initiation of antiretroviral therapy (ART) in HIV-positive partners of serodiscordant couples leads to rapid and sustained viral suppression, resulting in a 96% reduction in linked HIV transmission compared with delayed treatment [10]. This protective effect is mediated primarily through reductions in viral load, highlighting the biological and epidemiological basis for treatment as prevention.

Age has also been identified as an important determinant of viral load suppression among people living with HIV. Older patients are more likely to achieve and maintain suppression due to better adherence, greater health-seeking behavior, and more stable lifestyles, whereas adolescents and young adults often experience lower suppression rates due to adherence challenges, psychosocial barriers, and transitions from pediatric to adult care [11–13].

Similarly, gender differences influence viral suppression in serodiscordant couples. Women are more likely than men to achieve and sustain viral suppression, largely due to earlier engagement in care and higher adherence, whereas men often initiate ART later and exhibit lower adherence, which may increase transmission risk to uninfected partners [14–16].

Mathematical and statistical models have been an essential tools in exploring the dynamics of VL measures [17], understanding of the pathogenesis of HIV-1 infection and in assessment of the potency of antiretroviral therapies [18]. However, this can be challenging because of the potential intra-couple correlation and the presence of multiple measurements collected from the same study site or cluster, which therefore might not be independent. A second complication arises due to censoring (left, interval or right) of the measurements [19,20,21]. Censoring arises when the exact value of a measurement is not fully observed but is instead known to lie within a certain range. For the present study, left-censoring or Type I (left) censoring befalls when the measurement procedure has a LOD and observations fall below this limit, whereas interval-censoring arises when the measurement lies within an interval, i.e., between LOD and limit of quantitation (LOQ) instead of being observed exactly [22].

Dealing with outcomes that are left- and interval-censored may be more difficult because classical regression methods require a quantitative value for each observation [19,23] and parameter estimates obtained without accounting for censoring are expected to be biased. Ad hoc methods to deal with left-censored data are readily available and widely used as, e.g., substitution methods, imputation methods, and Tobit regression [20]. The simplest substitution method replaces a left-censored observation by the value LOD/2 (or LOD/$\sqrt{2}$) and an interval-censored observation by (LOD + LOQ)/2 and continues with the classical statistical methods.

Maximum likelihood estimation (MLE) based on, e.g., the log-normal distribution is often recommended as the appropriate approach [24] for estimating the geometric mean and geometric standard deviation for left-censored data. The MLE method produces estimators with required properties (consistency, asymptotic unbiasedness, and efficiency), and left- and interval-censoring can be naturally incorporated into parametric models [25,26]; therefore, maximum likelihood estimation (MLE) was chosen for this study, as it explicitly accounts for censoring within the likelihood function. This approach allows all observations, including censored values, to contribute appropriately to parameter estimation, thereby reducing bias in the estimation of means, variances, regression coefficients, and within-couple correlations, and ensuring inference targets the underlying VL distribution rather than values obtained through ad hoc substitution or truncation.

This work was motivated by VL data from the Mozambican national survey of prevalence, risk behavioural and information about immunization, Malaria and HIV/AIDS, the so-called IMASIDA 2015. Besides other health indicators, IMASIDA 2015 allowed to obtain population-based estimates of VL suppression among adults [27]. However, it was unclear how ART and age affect VL suppression among Mozambican couples. The analysis in this paper aims to investigate the association between age and log VL of woman and man within a couple, while accounting for the presence (yes/no) of antiretroviral biomarkers in blood. An additional question of interest is: how weakly or strongly are log VL of woman and man within a couple correlated, and does this correlation depend on covariates as well.

## 2 Methodology

In this section, we describe the IMASIDA 2015 survey design, variables and the proposed methods. In Sect 2.1, the survey design and variables are briefly described. Joint models for bivariate continuous outcomes are introduced in Sect 2.2. Next, this subsection describes how the censored (left and interval) nature of the two outcome variables was taken into account in the modeling process. Finally, we described the use of sample weights to account for the survey design.

### 2.1 IMASIDA design and variables description

The IMASIDA 2015 data were collected by a cross-sectional survey carried out by the National Institute of Health (INS), in collaboration with the National Bureau of Statistics of Mozambique (INE) between June 8 and September 20, 2015. Stratification and cluster sampling methods were used to ensure that inference at the provincial level was possible with nearly the same precision. Moreover, two-stage sampling was applied to access individuals within households. Enumeration Areas (EAs), households and individuals were Primary Sampling Units, Secondary Sampling Units and Tertiary Sampling Units, respectively. Under stratification sampling methods, the 11 provinces were considered as strata.

A total of 307 EAs were randomly sampled from 45,000 EAs; 134 EAs were selected from urban areas and 173 EAs from rural areas. A fixed number of households was systematically selected within each EA. Women and men aged between 15 and 59 years were eligible to participate in individual interviews and to provide a blood sample for HIV and VL tests. Analyses accounted for the IMASIDA 2015 survey design by applying sample weights reflecting its stratification, clustering, and three-stage sampling.

For each woman that was married or lived together with her male partner, an attempt was made to match her with her husband/partner using his household line number and a couple dataset was obtained. Laboratory-based testing was conducted for quantitative evaluation of viral load and qualitative detection of ARVs (atazanavir, lopinavir, efavirenz, and dolutegravir). For our analyses, we only used data from couples where both woman and man had long-term infection. By long-term infection, we refer to individuals with HIV VL of ≤ 1.000 copies/ml to the LAg Avidity EIA, individual specimens with an normalized optical density (ODn) ≥ 2.0 during initial testing, individual specimens with median ODn ≥ 1.5 and all individuals with ARV biomarkers in blood when median Lag ODn ≤ 1.525. All participants were tested for an of following ART used before 2014 in Mozambique: stavudine, lamivudine, nevirapine, zidovudine, efavirenz and tenofovir [28]. For details on the HIV testing algorithm in IMASIDA survey, we refer to [29]. The couple’s dataset from IMASIDA survey is public accessible at https://dhsprogram.com/what-we-do/survey/survey-display-467.cfm.

Table 1 shows a basic description of IMASIDA 2015 variables used in the analyses. The VL (log scale) of woman and man within a cohabiting couple are the two continuous outcomes of interest. The two variables were left-censored at 40 copies/ml and interval-censored between 40 and 550 copies/ml. Age for woman and man, ARV biomarkers for woman and man, CD4 cell counts for woman and man and residence area for the couple, were the independent variables included in the analyses.

**Table 1:** Basic description of IMASIDA variables used in the analyses.

Variable name	Type	Categories
VL (log scale) for man	Continuous	–
VL (log scale) for woman	Continuous	–
CD4 cell for man	Continuous	–
CD4 cell for woman	Continuous	–
Age for man	Continuous	–
Age for woman	Continuous	–
EA for couple	Continuous	–
ARV biomarkers for man	Binary	0: No (ref. category)
		1: Yes
ARV biomarkers for woman	Binary	0: No (ref. category)
		1: Yes
Residence area for couple	Binary	0: Rural (ref. category)
		1: Urban

Two-way interactions between covariates were considered. Heterogeneity across EAs was also investigated. To improve the convergence of the models and/or to avoid inflation of the standard errors, the ages of woman and man were centred, by subtracting their means. Of all variables included in the analyses, only the variable CD4 cell count had missing values (47.16%). Analyses accounted for the IMASIDA 2015 survey design by applying sample weights reflecting its stratification, clustering, and three-stage sampling.

### 2.2 Joint models for bivariate continuous outcomes

This section describes the joint marginal model and the approach to account for the left- and interval-censoring. Next, the model is extended with random-effects accounting for heterogeneity across EAs. Finally, the use of sample weights accounting for the survey design and the model building strategy are briefly described.

#### 2.2.1 Joint marginal model.

Let *y*_*M*_ and *y*_*W*_ be VL on log scale for woman and man within a couple. We assume that the pair $\left(y_{M},y_{W}\right)$ follows a bivariate normal distribution with mean $\left(\mu_{M},\mu_{W}\right)$, and variance $\left(\sigma_{M}^{2},\sigma_{W}^{2}\right)$, and within-couple correlation coefficient ρ. Let *x* denote a vector of covariates, possibly having an effect on any of the parameters.

Some values of *y*_*M*_ and *y*_*W*_ were left-censored at LOD (so $y_{M}\leq \text{LOD}$ and/or $y_{W}\leq \text{LOD})$ and/or were interval-censored between LOD and LOQ $(\text{LOD}\leq y_{M}\leq \text{LOQ}$ and/or $\text{LOD}\leq y_{W}\leq \text{LOQ})$. Thereby, the effect of covariates on the parameters can be modelled as follows (extending the classical linear regression model):


$$\mu_{M}=\beta_{1}x_{1}\;\mu_{W}=\beta_{2}x_{2}\;h_{3}(\sigma_{M})=\beta_{3}x_{3}\;h_{4}(\sigma_{W})=\beta_{4}x_{4}\;h_{5}(\rho )=\beta_{5}x_{5},$$ (1)

where *h*_3_, *h*_4_ and *h*_5_ are link functions. Appropriate choices are the log function for *h*_3_ and *h*_4_ (warranting that the estimated variance is always positive) and Fisher’s z-transformation log[(1+ρ)/(1−ρ)] for *h*_5_ (ensuring that the correlation is within the range (−1;1)). The vectors of regression coefficients $\beta_{1}$, ..., $\beta_{5}$ are estimating the effect of the covariates.

Model (1) can be embedded in different frameworks of estimation and inference, including the standard application of linear regression model.

To take into account the left- and interval-censored nature of the data, two censoring indicators, *C*_*M*_ for man’s VL and *C*_*W*_ for woman’s VL were defined and introduced into model components (1).

Let *C*_*M*_ = 2 when $y_{M}\leq LOD$, *C*_*M*_ = 1 if $LOD\leq y_{M}\leq LOQ$, and *C*_*M*_ = 0 otherwise. The same for woman, *C*_*W*_ = 2 when $y_{W}\leq LOD$, *C*_*W*_ = 1 if $LOD\leq y_{W}\leq LOQ$, and *C*_*W*_ = 0 otherwise.

There are 9 possible combinations for *y*_*M*_ and *y*_*W*_ that can be formulated as follows:

1. *C*_*M*_ = 0; *C*_*W*_ = 0: both VLs are uncensored
2. *C*_*M*_ = 0; *C*_*W*_ = 1: the man’s VL is uncensored while the woman’s VL is interval-censored
3. *C*_*M*_ = 0; *C*_*W*_ = 2: the man’s VL is uncensored while the woman’s VL is left-censored
4. *C*_*M*_ = 1; *C*_*W*_ = 0: the man’s VL is interval-censored while the woman’s VL is uncensored
5. *C*_*M*_ = 1; *C*_*W*_ = 1: both VLs are interval-censored
6. *C*_*M*_ = 1; *C*_*W*_ = 2: the man’s VL is interval-censored while the woman’s VL is left-censored
7. *C*_*M*_ = 2; *C*_*W*_ = 0: the man’s VL is left-censored while the woman’s VL is uncensored
8. *C*_*M*_ = 2; *C*_*W*_ = 1: the man’s VL is left-censored while the woman’s VL is interval-censored
9. *C*_*M*_ = 2; *C*_*W*_ = 2: both VLs are left-censored

For each of these 9 combinations, the likelihood function needs to be defined differently (see S1 Appendix A).

Table 2 shows the 9 combinations of types of censoring of VL values within the 352 couples included in the analysis. The majority of pairs are fully uncensored, more precisely 61.08% (215). About 14.77% (52) of the couples have both VL values left-censored. For 13.64% (48) of the pairs, the VL of the woman was left-censored and the VL for the man uncensored, whereas for 7.39% (26) of the pairs it was reversed. For only 11 couples there was at least one VL value interval-censored.

**Table 2:** Cross-classification of the type of censoring of the VL values for the 352 couples. LC = left-censored, IC = interval-censored, UN = uncensored.

	VL woman			
VL man	LC	IC	UN	Total
LC	52	2	26	80
IC	4	0	3	7
UN	48	2	215	265
Total	104	4	244	352

Although only 3.13% of the pairs have interval-censored VL values, and treating these interval-censored observations as left-censored would considerably simplify the methodology (resulting in only four combinations) with potentially limited impact on the analyses, we implement and illustrate the methodology using the full complexity of the data (nine combinations).

For the analysis, we will consider the VL values on log scale. Substituting the left-censored VL values with LOD/2 = 40/2 = 20 and the interval-censored VL values with (LOD + LOQ)/2=(550 + 40)/2 = 295, the mean and standard deviation of the log VL values for women are 7.80 and 3.28 respectively, and for men 8.54 and 3.15 respectively.

#### 2.2.2 Joint random-effects model.

Let *y*_*M*,*ij*_ and *y*_*W*,*ij*_ denote the log VL of woman and man, respectively, for couple *i* (*i* = 1,..., *n*) in EA *j* (*j* = 1,..., *N*). A total of 352 couples (*n* = 352) from 307 (*N* = 307) different EAs were extracted from the IMASIDA 2015 dataset and used in the analyses.

The model (1) for the mean log VL were then extended with random-effects at EA level to capture heterogeneity across EAs:


$$\begin{array}{c} \mu_{M,ij}=\beta_{1}x_{1,ij}+b_{M,j}\\ \mu_{W,ij}=\beta_{2}x_{2,ij}+b_{W,j}\\ \log\left(\sigma_{M,ij}\right)=\beta_{3}x_{3,ij}\\ \log\left(\sigma_{W,ij}\right)=\beta_{4}x_{4,ij}\\ \log[(1+\rho )/(1-\rho )]=\beta_{5}x_{5,ij}, \end{array}$$ (2)

where the random-effects *b*_*M*,*j*_ and *b*_*W*,*j*_ are assumed to be jointly normally distributed


$$(\begin{array}{c} b_{M,j}\\ b_{W,j} \end{array})\sim N_{2}\left(0,\Sigma_{EA}\right),$$ (3)

with zero mean vector and variance-covariance matrix


$$\begin{array}{c} \Sigma_{EA}=\left(\begin{array}{cc} \sigma_{EA_{M}}^{2} & \sigma_{EA_{M}}\sigma_{EA_{W}}\rho_{EA}\\ \sigma_{EA_{M}}\sigma_{EA_{W}}\rho_{EA} & \sigma_{EA_{W}}^{2} \end{array}\right).\; \end{array}$$ (4)

As in marginal Model (1), $\beta_{1}$,..., $\beta_{5}$ are unknown regression coefficients. All variables used in the analyses were measured at individual level (woman and man) except residence area which was measured at couple’s level.

The variances $\sigma_{EA_{M}}^{2}$ and $\sigma_{EA_{W}}^{2}$ quantify the degree of heterogeneity in the mean log VL values for women and men across EAs, and $\rho_{EA}$ relates to the correlation between the log VL values of a woman and a man living in the same EA.

The random-effects at EA level imply a second correlation structure: the log VL values of individuals living in the same EA are more alike than of individuals living in different EAs, so there is an “intra-EA correlation”. The full correlation structure can be expressed as follows.

Consider the correlation $\text{cor}(y_{M,ij},y_{W,i^{\prime }j^{\prime }})$ between the log VL value for a man of couple *i* living in EA *j* and the log VL value for a woman of couple *i*′ living in EA *j*′:


$$\rho_{MW}=\text{cor}(y_{M,ij},y_{W,i^{\prime }j^{\prime }})=$$

$$\begin{array}{c} \left\{ \begin{array}{cc} 0 & \text{different couples }i\neq \text{i' in different EAs }j\neq \text{j'}\\ & \;\\ \frac{\sigma_{EA_{M}}\sigma_{EA_{W}}\rho_{EA}}{\sqrt{\overset{2}{\underset{EA_{M}}{\sigma }}+\overset{2}{\underset{M}{\sigma }}}\sqrt{\overset{2}{\underset{EA_{W}}{\sigma }}+\overset{2}{\underset{W}{\sigma }}}} & \text{different couples }i\neq \text{i' in the same EA }j={\text{j}}^{\prime }\\ & \;\\ \frac{\sigma_{EA_{M}}\sigma_{EA_{W}}\rho_{EA}+\sigma_{M}\sigma_{W}\rho }{\sqrt{\overset{2}{\underset{EA_{M}}{\sigma }}+\overset{2}{\underset{M}{\sigma }}}\sqrt{\overset{2}{\underset{EA_{W}}{\sigma }}+\overset{2}{\underset{W}{\sigma }}}} & \text{same couple }i={\text{i}}^{\prime },\;implying\;\text{j}={\text{j}}^{\prime }\text{.} \end{array}\right.\; \end{array}$$ (5)

Note that it is assumed that woman and man of the same couple (*i* = *i*′) live in the same EA (*j* = *j*′), as the population of interest is that of cohabiting couples living in the same household. For the correlation $\text{cor}(y_{M,ij},y_{M,i^{\prime }j^{\prime }})$ between the log VL values of two different men *M* and *M*′ in EA *j* and *j*′, we have:


$$\rho_{MM^{\prime }}=\text{cor}(y_{M,ij},y_{M^{\prime },i^{\prime }j^{\prime }})=$$

$$\begin{array}{c} \left\{ \begin{array}{cc} 0 & \text{two men in different EAs }j\neq \text{j'}\\ & \;\\ \frac{\overset{2}{\underset{EA_{M}}{\sigma }}}{\sqrt{\overset{2}{\underset{EA_{M}}{\sigma }}+\overset{2}{\underset{M}{\sigma }}}\sqrt{\overset{2}{\underset{EA_{M}}{\sigma }}+\overset{2}{\underset{M^{\prime }}{\sigma }}}} & \text{two men in the same EA }j={\text{j}}^{\prime }. \end{array}\right. \end{array}$$ (6)

Here, it is assumed that two different men can only occur in two different couples $i\text{⧸}=i^{\prime }$. The same expression holds for the correlation $\rho_{WW}$ between the log VL values of two different women (just replace subscript *M* by subscript *W* in formula (6)).

The above general EA-level variance–covariance matrix $\Sigma_{EA}$ describes the joint distribution of male and female random effects within an enumeration area (EA), allowing both variance differences and correlation between partners. Specific values of the parameters simplify this structure:

1. **Shared random-effects model (** $\rho_{EA}=1$ **):**
  Setting $\rho_{EA}=1$ implies **perfect correlation** between the male and female random effects within an EA. In other words, knowing one random effect exactly determines the other up to a proportionality constant. Mathematically, this leads to the relation
  $$b_{M,j}=(\sigma_{EA_{M}}/\sigma_{EA_{W}})b_{W,j}$$
  so the two effects can be represented by a **single underlying EA-level effect**, reducing the number of free parameters.
2. **Independent random-effects model (** $\rho_{EA}=0$ **):**
  Setting $\rho_{EA}=0$ removes any association between male and female EA-level random effects. The effects are then **statistically independent**, and no proportionality constraint exists. This simplification reduces the model to two separate random effects per EA without any covariance parameter.
3. **Equal random-effects model (**$\sigma_{EA_{M}}=\sigma_{EA_{W}}$ **in the shared model):**
  Starting from the shared random-effects model, imposing equal variances for men and women ($\sigma_{EA_{M}}=\sigma_{EA_{W}}$) removes the proportionality factor, resulting in **identical random effects** for males and females within each EA:
  $$b_{M,j}=b_{W,j}$$

To select relevant covariates, a stepwise procedure was applied to the two univariate regression models for women and men separately. Effects of covariates on both parameters, mean and variance, were examined. Next, the significant covariates selected from the univariate analyses were used in the bivariate joint marginal and random-effects models.

In these joint models, the effect of covariates on the within-couple correlation parameter ρ was investigated, leading to models with constant and non-constant correlation ρ. The final joint model was selected using the Akaike Information Criterion (AIC) [30]. This criterion aims at finding the right balance between accuracy and simplicity (by penalizing for the number of parameters). Smaller values indicate a better fit of the model.

## 3 Results

### 3.1 Descriptive characteristics of the study sample

A total of 352 couples (women and men) were included in the analyses. Of these couples, about 64.80% (228) of them lived in rural area. Furthermore, 20.80% (73) and 11.80% (42) of total couples included in our analyzes, were from Nampula and Zambezia provinces, respectively. With regards of education level, 29.91% (105) of women had no formal education and 56.72% (200) had only primary education level. Of men, 63.63% (224) of them had primary level and 36.40% (128) had secondary level [31]. About 69.32% (244), 1.14% (4) and 29.55% (104) of the women had uncensored VL, interval-censored VL and left-censored VL, respectively, while 75.28% (265), 1.99% (7) and 22.73% (80) of the men had uncensored VL, interval-censored VL and left-censored VL, respectively. The correlation between the log VL values of couples is estimated as 0.380 (95% CI [0.257; 0.492]).

**Fig 1:**
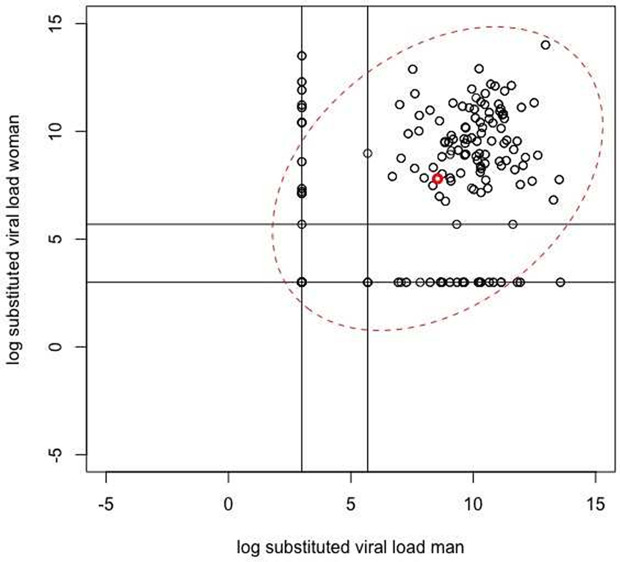
Scatter plot of the couples’ log VL pairs, together with (in red) the pair of averaged values and a two-dimensional 95% ellipse tracing a bivariate normal density contour. The horizontal and vertical lines are positioned at the log of the values LOD/2 and (LOD + LOQ)/2.

Fig 1 shows a scatter plot of the couples’ log VL pairs, together with (in red) the pair of averaged (substituted) values and a two-dimensional 95% ellipse tracing a bivariate normal density contour. The objective is to estimate the parameters of the bivariate normal distribution (on the log-scale) using maximum likelihood, appropriately accounting for the type of censoring, and investigating the effect of covariates on all parameters of the bivariate normal, including the correlation. Special interest goes to the effect of age and the effect of the ARV biomarkers status.

**Fig 2:**
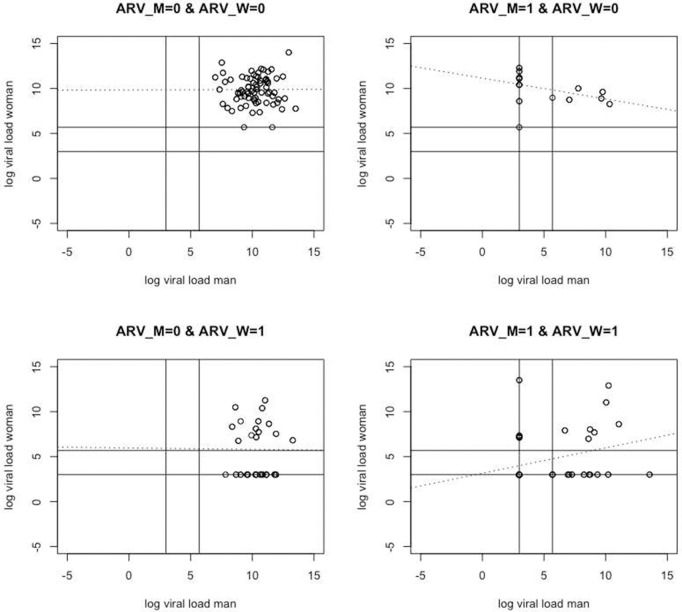
Scatter plot of the couples’ log VL pairs by ARV status of woman and man, together with a fitted linear regression line (dotted line). The horizontal and vertical lines are positioned at the log of the substituted values LOD/2 and (LOD + LOQ)/2.

Fig 2 shows the same type of scatter plot as Fig 1, but now separately for each combination of ARV status of woman and man. To visualize the correlation of the log VL values, a fitted regression line is shown as a dotted line. Some observations can be made regarding the effect of the ARV status. In case no ARV biomarkers were detected for both partners, no VL values were left-censored and the correlation of the (substituted) log VL values was about 0 (0.004 with 95% confidence interval (CI) [−0.16; 0.17]). In case the ARV status for both partners was positive, the VL values of both partners were partly left-censored. The correlation was 0.30 (95% CI [0.08; 0.49]).

In case the ARV status was only positive for the man, there were only left-censored VL values for the man. The correlation was now negative (−0.45 with 95% CI [−0.71; −0.08]). In case the ARV status was only positive for the woman, there were also only left-censored VL values for the woman. The correlation was about 0 (−0.007 with 95% CI [−0.28; 0.27]). So, the correlation seems to depend on the ARV statuses.

Table 3 shows means and standard deviations of the (substituted) log VL values for each of the four combinations of ARV biomarkers. As expected, it can be observed that the means of the log VL values are substantially lower when ARV = 1, for both sexes. The opposite seems to happen with the standard deviations, which are larger when ARV = 1, for both sexes. Question is how these descriptive insights are confirmed and extended by sound statistical analyses using maximum likelihood inference, while accounting appropriately for the design of the study, the censored nature of the data (instead of the ad hoc substitution) and for the effects of covariates.

**Table 3:** Means and standard deviations (sd) with respective standard errors (se), of the (substituted) log VL values for each of the four combinations of ARV biomarkers.

	VL woman		VL man	
Combination	mean(se)	sd(se)	mean(se)	sd(se)
$ARV_{M}=0,ARV_{W}=0$	9.89(0.12)	1.49(0.08)	10.28(0.12)	1.40(0.08)
$ARV_{M}=1,ARV_{W}=0$	9.93(0.29)	1.47(0.20)	5.80(0.53)	2.68(0.20)
$ARV_{M}=0,ARV_{W}=1$	6.13(0.37)	2.62(0.14)	10.36(0.17)	1.23(0.14)
$ARV_{M}=1,ARV_{W}=1$	5.17(0.30)	2.65(0.11)	5.85(0.31)	2.81(0.11)

se: standard error.

sd: standard deviation.

### 3.2 Joint marginal modeling and random-effects evaluation

Based on maximum likelihood and accounting for left- and interval-censoring, two univariate linear regression models were fitted for men and women separately, and a stepwise procedure was used to select significant factors affecting the mean and/or variance of log VL, for both univariate models. This exercise led to significant effects of the age and of ARV biomarkers for woman, and only age for man. Using these factors from the univariate analyses, the models for both sexes were fitted jointly marginally, and with different (multivariate) random-effects structures, capturing the heterogeneity across the EAs.

Table 4 compares the fit of all models: the joint marginal model without random EA effects and all random-effects models: equal, shared, independent and correlated random-effects models, with constant and non-constant correlation ρ. The column ‘-2ll’ shows the values of −2log(likelihood) (quality measure of fitting to the data at hand, the lower the better); the column ‘Parameters’ shows the number of parameters (#par, reflecting the complexity of the model) and the column ‘Rank’ refers to the ranking of the models according to the AIC criterion in the column ‘AIC’ (defined as −2log(likelihood)+2#par, balancing quality of fit with complexity and avoiding overfitting, the lower the AIC values, the better the model).

**Table 4:** Comparison of the marginal, equal, shared independent and correlated random-effects models with constant and non-constant ρ.

Model	ρ	−2ll	Par.	AIC	Rank_*AIC*_
Marginal model	Constant	1788.0	13	1814.0	10
Marginal model	Non-constant	1775.6	16	1807.6	6
Equal random-effects model	Constant	1780.1	14	1808.1	8
Equal random-effects model	Non-constant	1768.7	17	1802.7	2
Shared random-effects model	Constant	1778.7	15	1808.7	9
Shared random-effects model	Non-constant	1767.1	18	1803.1	3
Indep. random-effects model	Constant	1776.6	15	1806.6	5
**Indep. random-effects model**	**Non-constant**	**1766.3**	**18**	**1802.3**	**1**
Correlated random-effects model	Constant	1776.0	16	1808.0	7
Correlated random-effects model	Non-constant	1765.8	19	1803.8	4

The AIC criterion indicates that all non-constant correlation models are to be preferred above the corresponding versions with constant correlation. All random-effects models perform better than the marginal model without any EA random-effect. All random-effects models with non-constant correlation seem to fit essentially equally well, with AIC values ranging from 1802.3 to 1803.8; especially the best fitting Independent random-effects model and second best fitting Equal random-effects model (1802.3 and 1802.7 values respectively) are essentially indistinguishable.

That the model with independent EA effects and the model with equal EA effects are not distinguishable by AIC (and essentially the other random-effects models neither) is somewhat remarkable, at first sight. However, 83% of the EA’s have only one single couple in the sample, 16% of the EAs have two couples, and only about 1% of the EA’s have 3–7 couples in the sample (see Table 7 in the S3 Appendix C). This indicates there is very little to no information for estimating the $\rho_{EA}$ parameter, the main parameter differentiating the random-effects models. As formula (5) shows, this parameter also has to ‘compete’ with the ρ parameter, that measures the within-couple correlation across EAs. Table 8 in the S3 Appendix C also shows there is insufficient evidence in the data to reject $\rho_{EA}=0$ in the Correlated random-effects model (p-value = 0.479). Therefore, the Independent random-effects model is selected as the final model.

### 3.3 Inference for the independent random-effects model

The estimates of all 18 parameters of the Independent random-effects model model and their standard error estimates are shown in Table 5. There is no age effect on the mean log VL for men, but men with ARV biomarkers present have on average less values (−6.32 on log scale). The mean log VL for women and men with ARV biomarkers (estimates 10.26 and 10.02, respectively) are not statistically different (p-value = 0.166).

**Table 5:** Parameter and standard error estimates of the independent random-effects model with non-constant ρ.

*MC*	*int*	*age*	*ARV*_*M*_	*ARV*_*W*_	$ARV_{M}\times ARV_{W}$	$ARV_{W}\times age$
$\mu_{M}$	10.26(0.11)*	–	−6.32(0.74)*	–	–	–
$\mu_{W}$	10.02(0.13)*	−0.01(0.01)	–	−7.71(0.86)*	–	−0.21(0.09)*
$\log(\sigma_{M})$	0.12(0.08)	0.02(0.007)*	1.51(0.16)*	–	–	–
$\log(\sigma_{W})$	0.31(0.10)*	0.03(0.008)*	–	1.41(0.16)*	–	–
$\log(\sigma_{{EA}_{M}})$	-0.33(0.20)**	–	–	–	–	–
$\log(\sigma_{{EA}_{W}})$	-0.68(0.52)	–	–	–	–	–
$\log(\frac{1+\rho }{1-\rho })$	0.18(0.21)	–	−1.62(0.59)*	0.11(0.45)	2.16(0.82)*	–

* Significance at 5% level (Wald test)

** Significance at 5% level (mixture of $\chi_{0;1}$) for testing $\sigma_{{EA}_{M}}=0$

MC: model component.

int: intercept.

age: age for woman and man.

The interaction between age and ARV biomarkers appears to be only significant in the mean for women, showing that the effect of age on a woman’s log VL differs by ARV biomarkers presence. There is no significant effect of age in case of no ARV biomarkers, but it is negative in case the woman has ARV biomarkers in her blood. One additional year in age implies a decrease of −0.21 in mean log VL, for women with ARV biomarkers. For average-aged women (age about 33 years), having the ARV biomarkers present decreases the log VL with a value −7.71 (on average).

Note that exponentiated values of the average log VL do not refer to average VL values (on original scale), but rather to median VL values. And effects on the median VL are multiplicate rather than additive. So, for instance, on the original scale, we estimate the median VL for men without ARV biomarkers as exp(10.26)=28,567 copies/ml, whereas this median is exp(10.26−6.32)=51 copies/ml when the ARV biomarkers status is positive (a huge reduction of 1−exp(−6.32)=99.9%). For women with ARV biomarkers, any additional year of age decreases the median VL with a factor exp(−0.21)=0.811 (almost a reduction of 20%).

**Fig 3:**
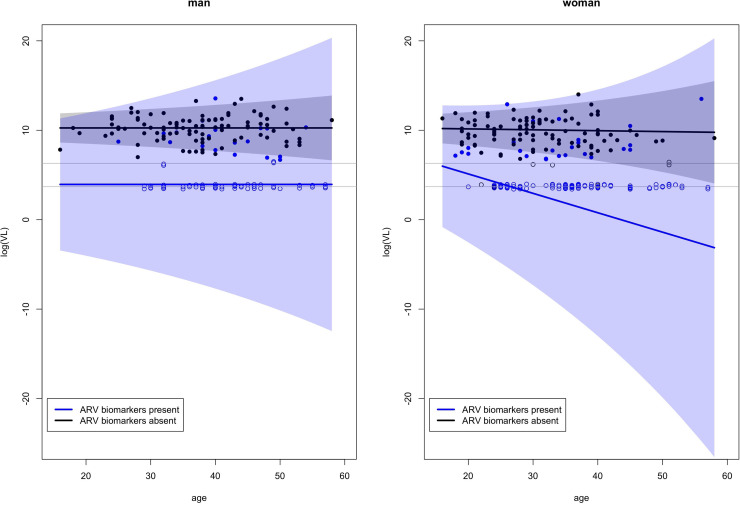
Scatter plots of log VL with fitted mean lines for men (left) and women (right), for ARV biomarkers present (blue) and absent (black). The shaded areas reflect the areas mean ± 2 × standard deviation of the fitted normal distributions. The horizontal grey lines at log(40) and log(550) locate the LOD and LOQ log values. The censored data (open bullets) at those LOD and LOQ values were scattered a bit to show their number more clearly.

For the standard deviations of the log VL values, Table 5 shows significant effects of age and ARV status, but no interaction effects, for both sexes. For both sexes, the standard deviation increases with age and ARV biomarkers presence (already observed in Table 3). Fig 3 shows, separately for woman and man and for ARV biomarkers present or absent, a scatter plot of the log VL data together with the characteristics of the fitted normal distribution (mean and mean ±2× standard deviation).

Regarding the EA random-effects, there was only evidence of such effect for men ($\sigma_{{EA}_{M}}=0$ rejected with p-value < 0.00). So, there is evidence for heterogeneity across EAs (or, equivalently, homogeneity within EAs) in the mean of the log VL among the male partners (while correcting for the effects of the fixed effects of Age and ARV), but insufficient evidence for such heterogeneity for the female partners. Note again that there are only a limited number of couples within the same EA (see Table 7 in S3 Appendix C), implying limited power for estimating and testing the variance components $\sigma_{{EA}_{M}}^{2}$ and $\sigma_{{EA}_{W}}^{2}$.

Only in case ARV biomarkers are present for the man, this correlation is significantly different from 0. If the ARV biomarkers are present for the woman, the correlation is positive, indicating that when the log VL for the male partner increases, the log VL for the female partner tends to increases as well. In case ARV biomarkers are only present for the man, the correlation is negative, indicating that when the log VL for the male partner increases, the log VL for the female partner tends to decrease.

**Fig 4:**
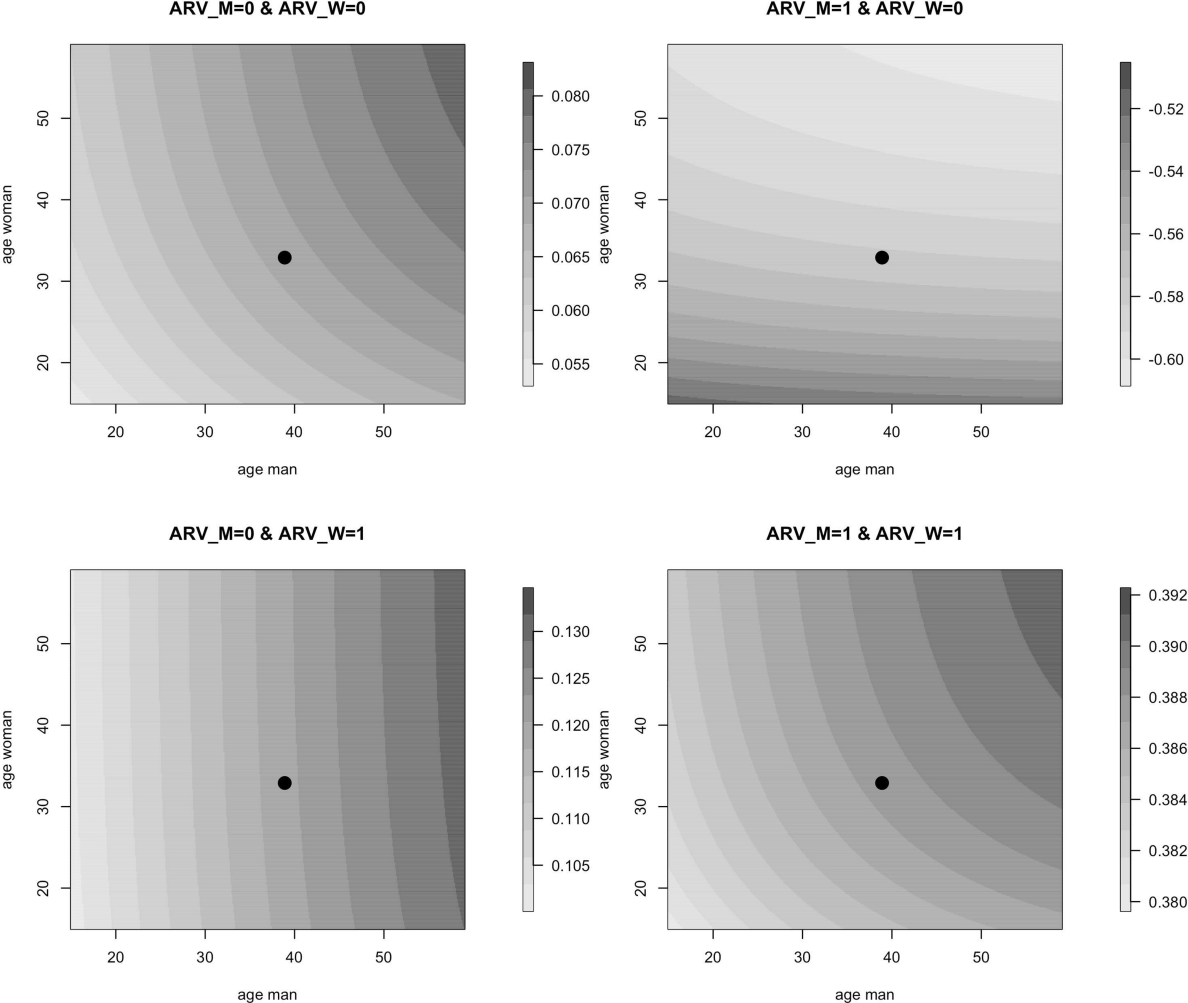
Correlation $\rho_{MW}$ as a function of age of the woman and age of the man, for all four combinations of ARV status. The black dot refers to the pair of average age of woman and man.

The image plots in Fig 4 show $\rho_{MW}$ as function of both ages, for all four ARV combinations. The darker the grey shade, the higher the correlation, within each panel’s scale. In case of ARV biomarkers absence for the man, the correlation is rather limited, ranging from 0.055 to 0.130. In case the ARV biomarkers are present for the man, the correlation $\rho_{MW}$ varies more substantially. If, in that case, the ARV biomarkers are not present for the woman, the correlation is negative across all ages, and increases in size mainly with the age of the woman, and much less with the age of the man. In case the ARV biomarkers are present for both partners, the correlation is always positive, increases with both ages, with highest correlation of about 0.40 for the highest ages for both partners.

The lower panel of Table 6 shows the correlation for two different men of average age and within the same EA. This correlation $\rho_{MM^{\prime }}$ is quite small, and decreases if one or both men had ARV biomarkers present. As the variance component $\sigma_{{EA}_{W}}$ is not significantly different from 0, this is neither the case for $\rho_{WW^{\prime }}$, and therefore no estimates are shown.

**Table 6:** Estimates for $\rho_{MW},\;\rho_{MM^{\prime }},\;\rho_{WW^{\prime }}$ for the four combinations of ARV biomarkers, at the averaged age for woman and man.

Cor	ARV Combination	estimate(se)	95% CI
$\rho_{MW}$	$ARV_{M}=0,ARV_{W}=0$	0.072(0.083)	(−0.093,0.236)
	$ARV_{M}=1,ARV_{W}=0$	-0.572(0.174)*	(−0.916,-0.228)
	$ARV_{M}=0,ARV_{W}=1$	0.122(0.168)	(−0.210,0.454)
	$ARV_{M}=1,ARV_{W}=1$	0.389(0.165)*	(0.062,0.715)
$\rho_{MM^{\prime }}$	$ARV_{M}=0,ARV_{M^{\prime }}=0$	0.288(0.101)*	(0.089,0.487)
	$ARV_{M}=1/0,ARV_{M^{\prime }}=0/1$	0.075(0.029)*	(0.016,0.133)
	$ARV_{M}=1,ARV_{M^{\prime }}=1$	0.019(0.009)*	(0.001,0.038)

* Significance at 5% level (Wald test).

## 4 Discussion and conclusions

This paper sought to investigate the association between age and VL of woman and man within couples, while accounting for the presence (yes/no) of ARV biomarkers in blood. In addition, this paper assessed how weak or strong the VL of woman and man within couples were correlated, and how this correlation depends on covariates. We assumed that the two response variables (log VL for woman and man) follow bivariate normal distribution, while accounting for the left- and interval-censored nature of the two VL values. Two univariate linear regression models were fitted for men and women separately. MLE was used while accounting for left- and interval-censoring nature of the outcomes. Stepwise procedure was used to select significant factors affecting the mean and/or variance of log VL, for both univariate models. This strategy led to significant effects of age and ARV biomarkers for women and only ARV for men. Later, the significant factors from the two univariate analyses, were used to fit joint marginal and random-effects model, capturing the heterogeneity across the EAs, assuming constant and non-constant correlation.

All non-constant correlation models were preferred to the corresponding versions with constant correlation. Next to that, all random-effects models with non-constant correlation fits better to the data. Was also remarkable that the model with independent EA effects and the model with equal EA effects (both with non-constant correlation) were not distinguishable by AIC. A weak positive correlation between the VL values of women and men within couples was observed. However, correlation’s estimates for the four combinations of ARV biomarkers, was positive only for couples where ARV biomarkers was detected in both sexes. This suggests a positive association such that lower male VL tends to be observed alongside lower female VL or vice versa.

Next, a negative correlation was estimated for pairs where the ARV biomarker was only detected in men, suggesting an inverse association between male and female VL in this subgroup or vice versa. No significant correlations between individuals within the same EA were observed across the four ARV biomarker combinations. In Mozambique, stavudine, lamivudine, and nevirapine were the most common regimens between 2004–2009. However, during 2010–2012, zidovudine, lamivudine, and nevirapine became more common, and between 2012–2013, increased tenofovir, lamivudine, and efavirenz use was noted for males, nonpregnant females, and pregnant females. During 2004–2013, stavudine use declined, whereas zidovudine and tenofovir use increased [28].

The effect of age on a woman’s VL changes by the ARV biomarkers status. No age effect on the mean VL for men was observed. This finding differs from other studies, where older HIV patients on ART were more likely to achieve viral suppression [32,33]. The possible explanation for this might be the difference in study period, study location, and sample size as well as the methodology used for data analysis. Furthermore, difference in viral suppression rate in general population, poor adherence and treatment failure, are other possible reasons [34].

IMASIDA 2015 survey reported higher VL suppression rate among women (37%) than men (22%) [31]. We did not find a difference in the median VL between women and men with ARV biomarkers. This finding is consistent with other studies that demonstrated similar rates of HIV treatment efficacy between male and female [35,36]. However, in South Africa it has been reported that higher odds of a detectable HIV VL were associated with being female [33]. Note that most of couples participated in IMASIDA survey were living in rural area, with little or no access to health services. In addition to that, majority of them had primary or no education level and limited awareness of the benefits of being viral suppressed.

Previous studies have reported that VL suppression rates are very low (1–7%) among PLHIV who are not receiving antiretroviral therapy (ART) [37–39]. A study conducted in Ethiopia identified poor treatment adherence, longer duration on ART, experience of drug toxicity, older age, and a recent CD4 count ≤200 cells/mm^3^ as factors associated with an increased risk of virologic failure [40]. Adherence has consistently been recognized as the most important determinant of VL among patients receiving ART [41,42].

Unfortunately, adherence and treatment failure indicators were not available in our study, as the IMASIDA 2015 survey did not collect this information. The findings of this study highlight the importance of understanding how ART use and age influence VL suppression within couples. These results may inform public health authorities in Mozambique in the continued design of programs that promote ART adherence among HIV-infected couples and emphasize ongoing VL monitoring as individuals age, particularly among women.

Future analyses could focus on modeling the association measure as the parameter of interest, as proposed by [43], while incorporating alternative assumptions for left- and interval-censored data.

## Supporting information

S1 Appendix A Combinations of types of censoring of log VL woman and man, and corresponding likelihood expressions. (PDF)

S2 Appendix B SAS code for the final independent random-effects model with non-constant ρ (see results in Table 5). (PDF)

S3 Appendix C Other tables. (PDF)
