# Supplementary material for: Joint modelling of left- and interval-censored viral load for couples in Mozambique
Source: PLoS One. 2026 Mar 30;21(3):e0345307. doi: 10.1371/journal.pone.0345307 (PMC13035130; doi:10.1371/journal.pone.0345307)
Supplement: S1 Appendix A — (PDF) [file pone.0345307.s001.pdf]

1. **Both VL man and VL woman are left-censored**

$$ll = F_{XY}(40,40) = \int_0^{40}\int_0^{40} f_{XY}(X,Y)d_X d_Y \tag{7}$$

2. **Both VL man and VL woman are interval-censored**

$$ll = F_{xy}(550,550)\text{-}F_{xy}(550,40) - F_{xy}(40,550) + F_{xy}(40,40)$$

$$= \int_0^{550}\int_0^{550} f_{xy}(x,y)d_x d_y - \int_0^{550}\int_0^{40} f_{xy}(x,y)d_x d_y - \int_0^{40}\int_0^{550} f_{xy}(x,y)d_x d_y$$

$$+ \int_0^{40}\int_0^{40} f_{xy}(x,y)d_x d_y \tag{8}$$

3. **VL man is left-censored while VL woman is interval-censored**

$$ll = F_{xy}(40,550) - F_{xy}(40,40) = \int_0^{40}\int_0^{550} f_{xy}(x,y)d_x d_y - \int_0^{40}\int_0^{40} f_{xy}(x,y)d_x d_y \tag{9}$$

4. **VL man is interval-censored while VL woman is left-censored**

$$ll = F_{xy}(550,40) - F_{xy}(40,40) = \int_0^{550}\int_0^{40} f_{xy}(x,y)d_x d_y - \int_0^{40}\int_0^{40} f_{xy}(x,y)d_x d_y \tag{10}$$

5. **Both VL man and VL woman are uncensored**

$$ll = \frac{1}{2\pi\sigma_x\sigma_y\sqrt{(1-\rho^2)}} exp\left[-\frac{\left[\frac{(x-\mu_x)}{\sigma_x}\right]^2 - 2\rho\left[\frac{(x-\mu_x)}{\sigma_x}\right]\left[\frac{(y-\mu_y)}{\sigma_y}\right] + \left[\frac{(y-\mu_y)}{\sigma_y}\right]^2}{2(1-\rho^2)}\right] \tag{11}$$

6. **VL man is uncensored and VL woman is left-censored**

$$ll = f_x(x)\phi\left[\frac{log(40) - \mu_y - \rho\frac{\sigma_y}{\sigma_x}(x-\mu_x)}{\sqrt{(1-\rho^2)\sigma_y}}\right] \tag{12}$$

7. **VL man is uncensored and VL woman is interval-censored**

$$ll = f_x(x)\left[\phi\left(\frac{log(550) - \mu_y - \rho\frac{\sigma_y}{\sigma_x}(x-\mu_x)}{\sqrt{(1-\rho^2)\sigma_y}}\right) - \phi\left(\frac{log(40) - \mu_y - \rho\frac{\sigma_y}{\sigma_x}(x-\mu_x)}{\sqrt{(1-\rho^2)\sigma_y}}\right)\right] \tag{13}$$

8. **VL man is left-censored and VL woman uncensored**

$$ll = f_y(y)\phi\left[\frac{log(40) - \mu_x - \rho\frac{\sigma_x}{\sigma_y}(y-\mu_y)}{\sqrt{(1-\rho^2)\sigma_x}}\right] \tag{14}$$

9. **VL man is internal-censored and VL woman uncensored**

$$ll = f_y(y)\left[\phi\left(\frac{log(550) - \mu_x - \rho\frac{\sigma_x}{\sigma_y}(y-\mu_y)}{\sqrt{(1-\rho^2)\sigma_x}}\right) - \phi\left(\frac{log(40) - \mu_x - \rho\frac{\sigma_x}{\sigma_y}(y-\mu_y)}{\sqrt{(1-\rho^2)\sigma_x}}\right)\right] \tag{15}$$
