# Supplementary material for: Joint modelling of left- and interval-censored viral load for couples in Mozambique
Source: PLoS One. 2026 Mar 30;21(3):e0345307. doi: 10.1371/journal.pone.0345307 (PMC13035130; doi:10.1371/journal.pone.0345307)
Supplement: S2 Appendix B — (PDF) [file pone.0345307.s002.pdf]

```
TITLE 'IND RANDOM EFFECT MODEL : BIVARIATE NORMAL DISTRIBUTION';
PROC NLMIXED DATA = MEN_WOMENALL8_1 NOAD QPOINTS = 25 MAXITER=10000;
PARMS B0_M = 10.2882  B2_M = -6.7435
      C0_M = 0.5120   C1_M = 0.02915  C2_M = 2.7830
      B0_W = 10.0719  B1_W = -0.00591 B2_W = -7.6178 B3_W = -0.2436
      C0_W = 0.7603   C1_W = 0.05049  C2_W = 2.6694
      COV0 = -0.01222 COV1 = -1.5184  COV2 = 0.2466 COV3 = 2.1000
      SM_EA = 0.03919 SW_EA = 7.2712;

MU_M = B0_M + B2_M*(ARV_MAN = 1) + EA_M;
MU_W = B0_W + B1_W*AGE_WOMAN_CENT + B2_W*(ARV_WOMAN = 1)
       + B3_W*AGE_WOMAN_CENT*(ARV_WOMAN = 1) + EA_W;

S2_M = EXP(C0_M + C1_M*AGE_MAN_CENT + C2_M*(ARV_MAN = 1)
 .  .  + C3_M*AGE_MAN_CENT*(ARV_MAN = 1));
S2_W = EXP(C0_W + C1_W*AGE_WOMAN_CENT + C2_W*(ARV_WOMAN = 1)
 .  .  + C3_W*AGE_WOMAN_CENT*(ARV_WOMAN = 1));

RHOETA = COV0 + COV1*(ARV_MAN = 1) + COV2*(ARV_WOMAN = 1)
         + COV3*(ARV_MAN = 1)*(ARV_WOMAN = 1);
RHO = (EXP(RHOETA)-1)/(EXP(RHOETA)+1);

/* THE VALUE OF PI */
PI = CONSTANT("pi");

/* COMPONENTS OF Z FOR PDF FUNCTION */
A = (LOG(VL_MAN)-MU_M)**2/S2_M;
B = 2*RHO*((LOG(VL_MAN)-MU_M)/SQRT(S2_M))*((LOG(VL_WOMAN)-MU_W)/SQRT(S2_W));
C = (LOG(VL_WOMAN)-MU_W)**2/S2_W;

/* CALCULATING THE VALUE OF Z FOR PDF FUNCTION */
Z = A - B + C;

/*1: Both VL man and VL woman are left-censored*/
IF (VL_CEN_MAN=2 AND VL_CEN_WOMAN=2) THEN
L = PROBBNRM(((LOG(40)-MU_M)/SQRT(S2_M)),((LOG(40)-MU_W)/SQRT(S2_W)),RHO);

/*2: Both VL man and VL woman are interval censored*/
IF (VL_CEN_MAN=1 AND VL_CEN_WOMAN=1) THEN L =
PROBBNRM(((LOG(550)-MU_M)/SQRT(S2_M)),((LOG(550)-MU_W)/SQRT(S2_W)),RHO)
-PROBBNRM(((LOG(550)-MU_M)/SQRT(S2_M)),((LOG(40)-MU_W)/SQRT(S2_W)),RHO)
-PROBBNRM(((LOG(40)-MU_M)/SQRT(S2_M)),((LOG(550)-MU_W)/SQRT(S2_W)),RHO)
 +PROBBNRM(((LOG(40)-MU_M)/SQRT(S2_M)),((LOG(40)-MU_W)/SQRT(S2_W)),RHO);

 /*3: VL man is left-censored while VL woman is interval-censored*/
IF (VL_CEN_MAN=2 AND VL_CEN_WOMAN=1) THEN
L =  PROBBNRM(((LOG(40)-MU_M)/SQRT(S2_M)),((LOG(550)-MU_W)/SQRT(S2_W)),RHO)
- PROBBNRM(((LOG(40)-MU_M)/SQRT(S2_M)),((LOG(40)-MU_W)/SQRT(S2_W)),RHO);

/*4: VL man is interval-censored while VL woman is left-censored*/
IF (VL_CEN_MAN=1 AND VL_CEN_WOMAN=2) THEN
L =  PROBBNRM(((LOG(550)-MU_M)/SQRT(S2_M)),((LOG(40)-MU_W)/SQRT(S2_W)),RHO)
-PROBBNRM(((LOG(40)-MU_M)/SQRT(S2_M)),((LOG(40)-MU_W)/SQRT(S2_W)),RHO);

/*5: Both VL man and VL woman are uncensored*/
IF (VL_CEN_MAN=0 AND VL_CEN_WOMAN=0) THEN
L = 1/(2*PI*SQRT(S2_M)*SQRT(S2_W)*SQRT(1-RHO**2))*EXP(-(Z/(2*(1-RHO**2))));

/*6: VL man is uncensored and VL woman is left-censored*/
```

```
IF (VL_CEN_MAN=0 AND VL_CEN_WOMAN=2) THEN
L = PDF("NORMAL",LOG(VL_MAN),MU_M,SQRT(S2_M))*PROBNORM((LOG(40)-MU_W
-RHO*SQRT(S2_W)/SQRT(S2_M)*(LOG(VL_MAN)-MU_M))/SQRT(S2_W*(1-RHO**2)));

/*7: VL man is uncensored and VL woman is interval-censored*/
IF (VL_CEN_MAN=0 AND VL_CEN_WOMAN=1) THEN
L = PDF("NORMAL",LOG(VL_MAN),MU_M,SQRT(S2_M))*(PROBNORM((LOG(550)-MU_W
-RHO*SQRT(S2_W)/SQRT(S2_M)*(LOG(VL_MAN)-MU_M))/SQRT(S2_W*(1-RHO**2)))
-PROBNORM((LOG(40)-MU_W-RHO*SQRT(S2_W)/SQRT(S2_M)*(LOG(VL_MAN)-MU_M))/
SQRT(S2_W*(1-RHO**2))));

/*8: VL man is left-censored and VL woman uncensored*/
IF (VL_CEN_MAN=2 AND VL_CEN_WOMAN=0) THEN
L = PDF("NORMAL",LOG(VL_WOMAN),MU_W,SQRT(S2_W))*PROBNORM((LOG(40)-MU_M
-RHO*SQRT(S2_M)/SQRT(S2_W)*(LOG(VL_WOMAN)-MU_W))/SQRT(S2_M*(1-RHO**2)));

/*9: VL man is internal-censored and VL woman uncensored*/
IF (VL_CEN_MAN=1 AND VL_CEN_WOMAN=0) THEN
L=PDF("NORMAL",LOG(VL_WOMAN),MU_W,SQRT(S2_W))*(PROBNORM((LOG(550)-MU_M
-RHO*SQRT(S2_M)/SQRT(S2_W)*(LOG(VL_WOMAN)-MU_W))/SQRT(S2_M*(1-RHO**2)))
-PROBNORM((LOG(40)-MU_M-RHO*SQRT(S2_M)/SQRT(S2_W)*(LOG(VL_WOMAN)-MU_W))/
SQRT(S2_M*(1-RHO**2))));

LL=LOG(L);
LL_WEIGHT=LL*WEIGHT;
MODEL LL_WEIGHT ~ GENERAL(LL_WEIGHT);
RANDOM EA_M EA_W~ NORMAL([0,0],[SM_EA, 0, SW_EA]) SUBJECT = EA;

/* SPECIFIC PARAMETERS OF INTEREST */
ESTIMATE 'LOG_SIGMA_M_INT' C0_M/2;
ESTIMATE 'LOG_SIGMA_M_AGE' C1_M/2;
ESTIMATE 'LOG_SIGMA_M_ARV' C2_M/2;

ESTIMATE 'LOG_SIGMA_W_INT' C0_W/2;
ESTIMATE 'LOG_SIGMA_W_AGE' C1_W/2;
ESTIMATE 'LOG_SIGMA_W_ARV' C2_W/2;

ESTIMATE 'LOG_SIGMA_EA_M' LOG(SQRT(SM_EA));
ESTIMATE 'LOG_SIGMA_EA_W' LOG(SQRT(SW_EA));

ESTIMATE 'SIGMA2_M0' EXP(C0_M);
ESTIMATE 'SIGMA2_M1' EXP(C0_M + C2_M);
ESTIMATE 'SIGMA2_W0' EXP(C0_W);
ESTIMATE 'SIGMA2_W1' EXP(C0_W + C2_W);
ESTIMATE 'RHO_00' (EXP(COV0) - 1)/(EXP(COV0) + 1);
ESTIMATE 'RHO_10' (EXP(COV0 + COV1) - 1)/(EXP(COV0 + COV1) + 1);
ESTIMATE 'RHO_01' (EXP(COV0 + COV2) - 1)/(EXP(COV0 + COV2) + 1);
ESTIMATE 'RHO_11' (EXP(COV0 + COV1 + COV2 + COV3) - 1)/(EXP(COV0 + COV1 + COV2 + COV3) + 1);
ESTIMATE 'RHO_MW_00' (SQRT(SIGMA2_M0*SIGMA2_W0)*RHO_00)/SQRT((SM_EA+SIGMA2_M0)*
(SW_EA+SIGMA2_W0));
ESTIMATE 'RHO_MW_10' (SQRT(SIGMA2_M1*SIGMA2_W0)*RHO_10)/SQRT((SM_EA+SIGMA2_M1)*
(SW_EA+SIGMA2_W0));
ESTIMATE 'RHO_MW_01' (SQRT(SIGMA2_M0*SIGMA2_W1)*RHO_01)/SQRT((SM_EA+SIGMA2_M0)*
(SW_EA+SIGMA2_W1));
ESTIMATE 'RHO_MW_11' (SQRT(SIGMA2_M1*SIGMA2_W1)*RHO_11)/SQRT((SM_EA+SIGMA2_M1)*
(SW_EA+SIGMA2_W1));
ESTIMATE 'RHO_MM_00' (SM_EA)/(SQRT((SM_EA+SIGMA2_M0)*(SM_EA+SIGMA2_M0)));
ESTIMATE 'RHO_MM_10' (SM_EA)/(SQRT((SM_EA+SIGMA2_M1)*(SM_EA+SIGMA2_M0)));
ESTIMATE 'RHO_MM_11' (SM_EA)/(SQRT((SM_EA+SIGMA2_M1)*(SM_EA+SIGMA2_M1)));
ESTIMATE 'RHO_WW_00' (SW_EA)/(SQRT((SW_EA+SIGMA2_W0)*(SW_EA+SIGMA2_W0)));
ESTIMATE 'RHO_WW_10' (SW_EA)/(SQRT((SW_EA+SIGMA2_W1)*(SW_EA+SIGMA2_W0)));
ESTIMATE 'RHO_WW_11' (SW_EA)/(SQRT((SW_EA+SIGMA2_W1)*(SW_EA+SIGMA2_W1)));

RUN;
```
