# Supplementary material for: Joint modelling of left- and interval-censored viral load for couples in Mozambique
Source: PLoS One. 2026 Mar 30;21(3):e0345307. doi: 10.1371/journal.pone.0345307 (PMC13035130; doi:10.1371/journal.pone.0345307)
Supplement: S3 Appendix C — (PDF) [file pone.0345307.s003.pdf]

Table 7. Number of couples per EA

# of couples	n(%)
1	291(82.67)
2	57(16.16)
3	1(0.28)
4	1(0.28)
5	1(0.28)
6	1(0.28)
7	1(0.28)

Table 8. Parameter and standard error estimates of marginal, equal, shared, independent and correlated random-effects model with constant ρ.

			**Marginal model**			
MC	$int.$	age	ARV_M	ARV_W	$ARV_M \times ARV_W$	$ARV_W \times age$
μ_M	10.26(0.09)*	-	-6.70(0.79)*	-	-	-
μ_W	10.05(0.13)*	-0.01(0.01)	-	-7.62(0.85)*	-	-0.24(0.09)*
$\log(\sigma_M)$	0.28(0.05)*	0.02(0.005)*	1.37(0.15)*	-	-	-
$\log(\sigma_W)$	0.39(0.06)*	0.03(0.01)*	-	1.33(0.15)*	-	-
$\log(\frac{1+\rho}{1-\rho})$	0.17(0.14)	-	-	-	-	-
			**Equal random-effects model**			
μ_M	10.26(0.10)*	-	-6.82(0.80)*	-	-	-
μ_W	10.05(0.14)*	-0.01(0.01)	-	-7.67(0.85)*	-	-0.26(0.09)*
$\log(\sigma_M)$	0.18(0.07)*	0.02(0.006)*	1.47(0.16)*	-	-	-
$\log(\sigma_W)$	0.31(0.07)*	0.03(0.007)*	-	1.41(0.15)*	-	-
$log(\sigma_{EA})$	-0.59(0.21)**	-	-	-	-	-
$\log(\frac{1+\rho}{1-\rho})$	-0.13(0.17)	-	-	-	-	-
			**Shared random-effects model**			
μ_M	10.26(0.11)*	-	-6.74(0.79)*	-	-	-
μ_W	10.05(0.13)*	-0.01(0.01)	-	-7.60(0.85)*	-	-0.25(0.09)*
$\log(\sigma_M)$	0.12(0.08)*	0.02(0.007)*	1.51(0.16)*	-	-	-
$\log(\sigma_W)$	0.37(0.07)*	0.03(0.007)*	-	1.34(0.15)*	-	-
$log(\sigma_{EA})$	-0.33(0.20)	-	-	-	-	-
ψ	0.34(0.40)	-	-	-	-	-
$\log(\frac{1+\rho}{1-\rho})$	0.01(0.23)	-	-	-	-	-
			**Independent random-effects model**			
μ_M	10.26(0.11)*	-	-6.67(0.78)*	-	-	-
μ_W	10.03(0.14)*	-0.01(0.02)	-	-7.65(0.85)*	-	-0.23(0.09)*
$\log(\sigma_M)$	0.12(0.08)	0.02(0.007)*	1.51(0.16)*	-	-	-
$\log(\sigma_W)$	0.28(0.10)*	0.03(0.008)*	-	1.44(0.16)*	-	-
$log(\sigma_{EA_M})$	-0.33(0.20)	-	-	-	-	-
$log(\sigma_{EA_W})$	-0.43(0.34)	-	-	-	-	-
$\log(\frac{1+\rho}{1-\rho})$	0.16(0.16)	-	-	-	-	-
			**Correlated random-effects model**			
μ_M	10.26(0.11)*	-	-6.73(0.79)*	-	-	-
μ_W	10.04(0.14)*	-0.01(0.02)	-	-7.69(0.86)*	-	-0.25(0.09)*
$\log(\sigma_M)$	0.11(0.08)	0.02(0.007)*	1.52(0.16)*	-	-	-
$\log(\sigma_W)$	0.26(0.10)*	0.03(0.008)*	-	1.45(0.16)*	-	-
$log(\sigma_{EA_M})$	-0.32(0.19)	-	-	-	-	-
$log(\sigma_{EA_W})$	-0.40(0.32)	-	-	-	-	-
COV_{EA}	0.13(0.17)	-	-	-	-	-
$\log(\frac{1+\rho}{1-\rho})$	0.07(0.20)	-	-	-	-	-

* Significance at 5% level (Wald test)
** Significance at 5% level (Mixture of $\chi_{0;1}$)
MC: model component
int: intercept
age: age for woman and man

Table 9. Parameter and standard error estimates of marginal, equal, shared, independent and correlated random-effects model with non-constant ρ.

MC	$int.$	age	ARV_M	ARV_W	$ARV_M \times ARV_W$	$ARV_W \times age$
**Marginal model**						
μ_M	10.26(0.09)*	-	-6.38(0.75)*	-	-	-
μ_W	10.03(0.13)*	-0.01(0.01)	-	-7.68(0.87)*	-	-0.21(0.09)*
$\log(\sigma_M)$	0.28(0.05)*	0.02(0.006)*	1.38(0.15)*	-	-	-
$\log(\sigma_W)$	0.39(0.06)*	0.03(0.006)*	-	1.33(0.15)*	-	-
$\log(\frac{1+\rho}{1-\rho})$	0.18(0.16)	-	-1.84(0.53)*	0.15(0.40)	2.29(0.76)*	-
**Equal random-effects model**						
μ_M	10.25(0.10)*	-	-6.35(0.76)*	-	-	-
μ_W	10.03(0.14)*	-0.01(0.01)	-	-7.78(0.87)*	-	-0.22(0.09)*
$\log(\sigma_M)$	0.18(0.07)*	0.02(0.007)*	1.47(0.16)*	-	-	-
$\log(\sigma_W)$	0.31(0.07)*	0.03(0.007)*	-	1.41(0.15)*	-	-
$log(\sigma_{EA})$	-0.56(0.21)**	-	-	-	-	-
$\log(\frac{1+\rho}{1-\rho})$	-0.27(0.24)	-	-1.09(0.61)	0.44(0.44)	1.71(0.79)*	-
**Shared random-effects model**						
μ_M	10.25(0.11)*	-	-6.32(0.75)*	-	-	-
μ_W	10.03(0.13)*	-0.01(0.01)	-	-7.68(0.86)*	-	-0.21(0.09)*
$\log(\sigma_M)$	0.12(0.08)*	0.02(0.007)*	1.52(0.16)*	-	-	-
$\log(\sigma_W)$	0.38(0.07)*	0.03(0.007)*	-	1.34(0.15)*	-	-
$log(\sigma_{EA})$	-0.33(0.20)	-	-	-	-	-
ψ	0.29(0.50)	-	-	-	-	-
$\log(\frac{1+\rho}{1-\rho})$	-0.01(0.39)	-	-1.52(0.73)*	0.25(0.51)	2.10(0.88)*	-
**Independent random-effects model**						
μ_M	10.26(0.11)*	-	-6.32(0.74)*	-	-	-
μ_W	10.02(0.13)*	-0.01(0.02)	-	-7.71(0.86)*	-	-0.21(0.09)*
$\log(\sigma_M)$	0.12(0.08)	0.02(0.007)*	1.51(0.16)*	-	-	-
$\log(\sigma_W)$	0.31(0.10)*	0.03(0.007)*	-	1.41(0.16)*	-	-
$\log(\sigma_{EA_M})$	-0.33(0.20)**	-	-	-	-	-
$\log(\sigma_{EA_W})$	-0.68(0.52)	-	-	-	-	-
$\log(\frac{1+\rho}{1-\rho})$	0.18(0.21)	-	-1.62(0.59)*	0.11(0.45)	2.16(0.82)*	-
**Correlated random-effects model**						
μ_M	10.25(0.11)*	-	-6.31(0.75)*	-	-	-
μ_W	10.03(0.14)*	-0.01(0.01)	-	-7.73(0.86)*	-	-0.21(0.09)*
$\log(\sigma_M)$	0.11(0.08)	0.02(0.007)*	1.51(0.16)*	-	-	-
$\log(\sigma_W)$	0.30(0.10)*	0.03(0.007)*	-	1.42(0.16)*	-	-
$log(\sigma_{EA_M})$	-0.32(0.19)	-	-	-	-	-
$log(\sigma_{EA_W})$	-0.59(0.45)**	-	-	-	-	-
COV_{EA}	0.13(0.19)	-	-	-	-	-
$\log(\frac{1+\rho}{1-\rho})$	0.02(0.30)	-	-1.41(0.66)*	0.23(0.48)	1.98(0.84)*	-

* Significance at 5% level (Wald test)
** Significance at 5% level (Mixture of $\chi_{0;1}$)
MC: model component
int: intercept
age: age for woman and man
